# Longitudinal Changes in Milk Microorganisms in the First Two Months of Lactation of Primiparous and Multiparous Cows

**DOI:** 10.3390/ani13121923

**Published:** 2023-06-08

**Authors:** Huan Zhu, Renfang Miao, Xinxu Tao, Jianhao Wu, Licheng Liu, Jiachen Qu, Hongzhi Liu, Yanting Sun, Lingyan Li, Yongli Qu

**Affiliations:** 1 Heilongjiang Key Laboratory of Efficient Utilization of Feed Resources and Nutrition Manipulation in Cold Region, College of Animal Science and Veterinary Medicine, Heilongjiang Bayi Agricultural University, No. 5 Xinyang Road, Daqing 163319, China; zhuhuan1982@sina.com (H.Z.); M_Leslie@163.com (R.M.); txx09090416@163.com (X.T.);; 2 Key Laboratory of Low-Carbon Green Agriculture in Northeastern China, Ministry of Agriculture and Rural Affairs P. R. China, Heilongjiang Bayi Agricultural University, No. 5 Xinyang Road, Daqing 163319, China; 3 College of Science, Heilongjiang Bayi Agricultural University, No. 5 Xinyang Road, Daqing 163319, China; 4 Bright Farming Co., Ltd., No. 1518, West Jiangchang Road, Shanghai 200436, China; wujianhao@brightdairy.com; 5 Institute of Animal Husbandry and Veterinary Medicine, Heilongjiang Academy of Agricultural Reclamation, No. 101 Xiangfu Road, Herbin 150038, China; lichengliu2023@163.com; 6 School of Civil Engineering, Xi’an University of Architecture & Technology, No. 99 Yanta Road, Xi’an 710064, China; sunyanting98@163.com

**Keywords:** cow milk, lactation stage, milk microbiota, diversity, parity

## Abstract

**Simple Summary:**

Our results demonstrated the dynamic changes in cow milk microbiota over the first two months of lactation, and the differences in microbial composition between primiparous cows and multiparous cows. The presence and fluctuation of some typically gut-associated microbes in milk indicated that the metabolic changes in dairy cows may influence the microbial structure of cow milk. These changes may potentially be correlated with milk components.

**Abstract:**

The present experiment was carried out to analyze the longitudinal changes in milk microorganisms. For this purpose, milk samples were collected from 12 healthy cows (*n* = 96; six primiparous cows and six multiparous cows) at eight different time points. The characteristics and variations in microbial composition were analyzed by 16S rRNA gene high-throughput sequencing. In the primiparous group, higher and more stable alpha diversity was observed in transitional and mature milk compared with the colostrum, with no significant difference in alpha diversity at each time point in the multiparous group. Proteobacteria, Firmicutes, Bacteroidota, and Actinobacteriota were the most dominant phyla, and *Pseudomonas*, *UCG-005*, *Acinetobacter*, *Vibrio*, *Lactobacillus*, *Bacteroides*, *Serratia*, *Staphylococcus*, and *Glutamicibacter* were the most dominant genera in both primiparous and multiparous cow milk. Some typically gut-associated microbes, such as *Bacteroides*, *UCG-005*, and *Rikenellaceae_RC9_gut_group*, etc., were enriched in the two groups. Biomarker taxa with the day in time (DIM) were identified by a random forest algorithm, with *Staphylococcus* showing the highest degree of interpretation, and the difference in milk microbiota between the two groups was mainly reflected in 0 d–15 d. Additionally, network analysis suggested that there were bacteria associated with the total protein content in milk. Collectively, our results disclosed the longitudinal changes in the milk microbiota of primiparous and multiparous cows, providing further evidence in dairy microbiology.

## 1. Introduction

Milk is a complex and species-specific biological fluid containing immune cells and various active molecules such as sugars, nucleotides, lipids, immunoglobulins, antibacterial proteins, cytokines, and other immune regulatory factors. Milk aims to satisfy the nutritional requirements of the mammalian offspring and plays numerous functional roles in the development of offspring [1,2,3].

Recently, studies based on 16S rRNA high-throughput sequencing have analyzed the microbial community structure in milk, showing rich microbial communities in milk [4,5]. The appropriate temperature conditions and rich nutrients in the udder are conducive to the growth of microorganisms [6]. More research has been performed on dairy cow milk microorganisms, but most of them focused on a cross-sectional description of the microbiome. Oikonomou et al. [7,8] found that every healthy sample contained four bacterial genera (*Faecalibacterium* spp., *unclassified Lachnospiraceae*, *Propionibacterium* spp., and *Aeribacillus* spp.). Addis et al. [1] summarized the most abundant genera in milk wer *Ralstonia*, *Pseudomonas*, *Sphingomonas*, *Stenotrophomonas*, *Psychrobacter*, *Bradyrhizobium*, *Corynebacterium*, *Pelomonas*, and *Staphylococcus*.

Compared with mature milk, colostrum harbored a rich and diverse microbial community, the three most common genera being *Staphylococcus*, *Prevotella*, and *Pseudomonas*. This microbial community was conducive to intestinal flora colonization and nutrient acquisition in the calf [9,10]. Lima et al. [10] also reported differences in the microbial community structure related to parity, with a richer colostrum microbiota in primiparous cows compared to multiparous cows. In addition to that, the existing research results indicated that there were differences in the microbial diversity in the uterus, vagina, and rumen between primiparous cows and multiparous cows [11,12,13,14]. We speculated that changes in cows after first calving might lead to the differences in microorganisms in different parts, but this hypothesis should be studied further. Niyazbekova et al. [15] collected colostrum and mature milk samples at different time points, and the results showed significant changes in the composition of milk microbiota in the lactation stage, with *Proteobacteria*, *Firmicutes*, *Actinobacteria*, and *Bacteroidetes* being the predominant phyla in both colostrum and mature milk. Although some cross-sectional studies have investigated bacterial flora in milk, additional longitudinal studies should be performed to elucidate the complex and potentially dynamic microbiology of milk in cows.

In view of this, we hypothesize that the milk composition in cow milk shifts throughout the day in time (DIM), and the milk microbiota differences are present between primiparous cows and multiparous cows. To verify this hypothesis, we obtained milk samples from the two groups of cows for 16S rRNA sequencing analysis. Additionally, the potential correlation between milk components and microbiota was investigated. This study might a suitable basis for elucidating the potential synergistic interactions between microbiota in future.

## 2. Materials and Methods

### 2.1. Experimental Animals

This study was approved by the Animal Welfare and Ethics Committee of Heilongjiang Bayi Agriculture University (Daqing, China), and followed the regulations on the Administration of Experimental Animal Affairs (State Science and Technology Commission, Beijing, China, 1988).

Healthy Holstein cows were procured at the dairy farm of Heilongjiang Academy of Agricultural Sciences (Heihe, China) from November to December 2021. A total of 30 candidate healthy cows were selected at the beginning of the sampling period. A candidate cow would be excluded if its SCC was greater than 20 × 10^5^ (mL), or it was suffering from any other illness during the sampling period. Finally, 6 primiparous cows (PC) and 6 multiparous cows (MC) were selected. None of these cows required antibiotics during the sampling period. Cows were provided with normal feeding materials. The ingredients and nutritional composition of diets and forages are shown in Table 1.

### 2.2. Sample Collection

Milk samples (*n* = 96) were collected from each cow on parturition day (0 d), 3 days (3 d), 5 days (5 d), 7 days (7 d), 15 days (15 d), 21 days (21 d), 30 days (30 d), and 60 days (60 d) postpartum. Before collecting the milk samples, the udder surface of each cow was cleaned with sterile water and then scrubbed with 75% ethanol, and the milk samples were collected into two sterile tubes after discarding the first few drops of milk. Aside from the colostrum samples on day 0 that were collected within 1 h after delivery, the transition milk and mature milk samples were collected between 9:00 am and 11:00 am at each sampling day. Each sampling was conducted within 1 h before milking the cows. The samples used for the milk component analysis were added with potassium dichromate and stored at 4 °C, and the rest of the samples for 16S rRNA sequencing were temporarily stored in liquid nitrogen and were transferred to the laboratory and frozen to −80 °C in a refrigerator.

### 2.3. DNA Extraction and Amplification of V4 Regions of 16S rRNA Gene

After thawing the frozen samples at room temperature, bacterial genomic DNA was extracted by the cetyltrimethylammonium bromide (CTAB) method [14]. Agarose gel electrophoresis was used to assess the purity and concentration of DNA. Then suitable amounts of sample DNA were taken and diluted to 1 ng/µL by using sterile water. Subsequently, 1% agarose gel electrophoresis was used to detect the integrity of the DNA, and both the purity and concentration of each DNA sample were measured by a NanoDrop 2000 spectrophotometer (Thermo Fisher Scientific, Waltham, MA, USA). The isolated DNA was frozen at −20 °C until the next experiment. The V4 region of the 16S rRNA was amplified by using the primers 515F (5′-GTGCCAGCMGCCGCGGTAA-3′) and 806R (5′-GGACTACHVGGGTWTCTAAT-3′). The target bands were recovered using a gel recovery kit provided by Qiagen, and a TruSeq^®^ DNA PCR-Free Sample Preparation Kit was used for amplicon library preparation. PCR reagents and conditions were the same as those described by Winther et al. [16]. The final library concentration was quantified using Qubit 2.0 (ThermoFisher Scientific, Waltham, MA, USA) and quantitative PCR, and the sequencing was on a NovaSeq6000 platform (Illumina, San Diego, CA, USA).

### 2.4. Bioinformatical and Statistical Analysis

The sequence data from each sample was separated based on the PCR amplification primer sequence and barcode. In order to obtain the raw lags, FLASH software (V1.2.7, http://ccb.jhu.edu/software/FLASH/, (accessed on 9 February 2022) [17] was applied to assemble reads without the barcode and primer [18]. Low-quality tags were filtered out by following the QIIME software (V1.9.1) guidelines [19]. The tags were compared with the species annotation database to detect and remove chimera sequences. Then, effective tags were obtained and clustered to operational taxonomic units (OTUs) based on 97% identity of the sequences by Uparse software (V7.0.1001) [20,21]. The representative OTUs with the highest frequencies of occurrence for taxonomic information were selected and annotated based on the Mothur method and the SSUrRNA database of SILVA132 [22,23].

With the QIIME software (V1.9.1), alpha and beta diversity were analyzed, and the alpha index was evaluated by an ANOVA test. The community diversity, total number of species, and the observed OTUs were analyzed by the Shannon, Chao 1, and Observed-species indices. Beta diversity was assessed by weighted and unweighted UniFrac and Bray-Curtis distance, and visualized by Principal Coordinate Analysis (PCoA). Molecular variation analysis of variance (AMOVA) was performed to calculate the statistical significance of the spatial structure of the PCoA plots. A Tukey HSD test was conducted to analyze the variations in dominant genera at different time points, and the Wilcoxon test was performed to identify the differences in abundance in dominant genera between the two groups for each time point. The cut-off confidence level of all statistical analyses was 95%. A random forest model was established using the R package “randomForest” with 10-fold cross-validation and five repeats to identify the lists of taxa ranked by DIM. In the co-occurrence network interface, Spearman’s rank correlation was used to investigate the relationships between the milk components and predominant genera. Strong and significant correlations were defined as *r* < −0.6 or *r* > 0.6, *p* < 0.05. All graphical presentations were drawn with R software (V4.0.3).

### 2.5. Milk Components Analysis

The milk components were analyzed using MilkoScan FT1 (FOSS, Copenhagen, Denmark), including milk fat, total protein, and urea nitrogen.

## 3. Results

### 3.1. Alpha Diversity of the Milk Microbiome Community in Primiparous and Multiparous Cows

In this study, milk samples (*n* = 96) were collected from 6 primiparous cows and 6 multiparous cows at eight time points (0 d, 3 d, 5 d, 7 d, 15 d, 21 d, 30 d, and 60 d) to explore longitudinal changes in microbiome diversity. Due to taxonomic classification, samples that failed quality control were excluded, and the remaining 93 samples were included in the analysis. We obtained 5,982,976 high-quality bacterial sequences at the genus level, among which 3,067,048 reads belonged to the PC group, and the remaining reads belonged to the MC group.

**Figure 1:**
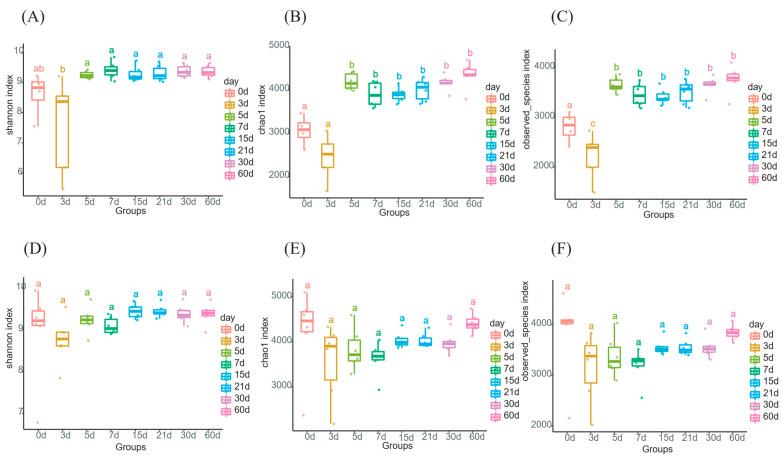
Time series variation of alpha diversity indices. (**A**–**C**) Diversity indices Shannon, Chao1, and Observed-species in the PC group; (**D**–**F**) Diversity indices Shannon, Chao1, and Observed-species in the MC group. Different letters denote significant differences among groups tested by Tukey HSD test. Significance level: *p* < 0.05; test method: Tukey HSD test.

In order to determine the richness and diversity of the milk microbiome, the indices of alpha diversity (Shannon, Chao 1, and observed-species) were calculated during the sampling duration. These indices on 3 d were significantly lower than that of the following days (Figure 1A–C), but no significant difference in these indices was observed in all sampling time points in the MC group (Figure 1D–F). Additionally, there were no significant differences in all alpha diversity indexes between the two groups at each time point. The gradual stabilization of Shannon diversity curves indicated that the sequencing depth was sufficient to represent a representative microbiome diversity (Figure S1). All the indices are listed in Supplementary Table S1.

### 3.2. Beta Diversity of the Milk Microbiome Community in Primiparous and Multiparous Cows

**Figure 2:**
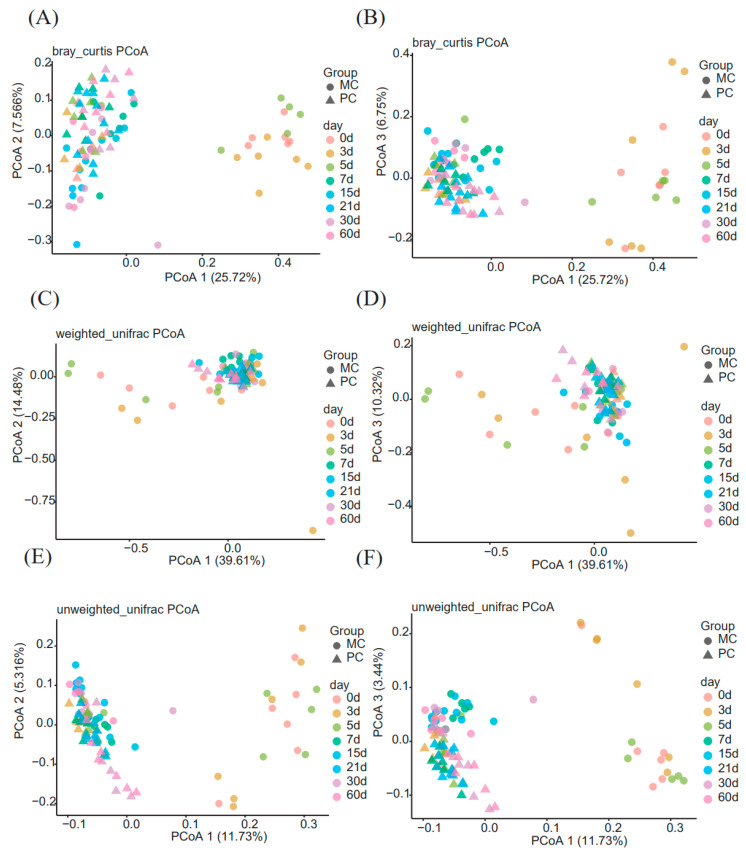
Time-series analysis of beta diversity. (**A**) PCoA based on Bray-Curtis (Axis 1 and Axis 2); (**B**) PCoA based on Bray-Curtis (Axis 1 and Axis 3); (**C**) PCoA based on weighted_unifrac distances (Axis 1 and Axis 2); (**D**) PCoA based on weighted_unifrac distances (Axis 1 and Axis 3); (**E**) PCoA based on unweighted_unifrac distances (Axis 1 and Axis 2); (**F**) PCoA based on unweighted_unifrac distances (Axis 1 and Axis 3). PC: primiparous cows; MC: multiparous cows.

Beta diversity was estimated by the weighted and unweighted UniFrac and Bray-Curtis distance to illustrate the dynamic changes in the milk microbiota in the two groups using the PCoA plots (Figure 2) from colostrum (0 d) to transitional milk (3 d), and then to mature milk (after 5 d). The PCoA plots showed obvious clustering of the milk microbiota community in the first coordinate axis from 0 d to 5 d in the MC group (Figure 2), indicating a convergence of the milk microbiota over time. The lactation time and parity were the main factors influencing the milk microbiota composition.

AMOVA based on weighted distance was used to evaluate the statistically significant differences in spatial separation as a DIM function in the PCoA plots. As seen in Figure S2, significant differences in milk microbiota diversity were observed in the PC group as DIM increased (*p* < 0.05), with the exception of similarities between 0 d and 3 d (*p* = 0.067), 3 d and 5 d (*p* = 0.122), 3 d and 7 d (*p* = 0.184), 5 d and 7 d (*p* = 0.724), 7 d and 21 d (*p* = 0.056), 15 d and 30 d (*p* = 0.051), 21 d and 30 d (*p* = 0.085), and 3 d and 60 d (*p* = 0.487). In the MC group, significant differences in milk microbiota diversity were observed between different time points (*p* < 0.05), with the exception of similarities between 0 d and 3 d (*p* = 0.341), 3 d and 60 d (*p* = 0.055), 15 d and 21 d (*p* = 0.27), 15 d and 30 d (*p* = 0.357), 15 d and 60 d (*p* = 0.242), 21 d and 30 d (*p* = 0.912), days 21 d and 60 d (*p* = 0.325), and 30 d and 60 d (*p* = 0.721).

### 3.3. Composition Changes in the Milk Microbiome Community with DIM in Primiparous and Multiparous Cows

**Figure 3:**
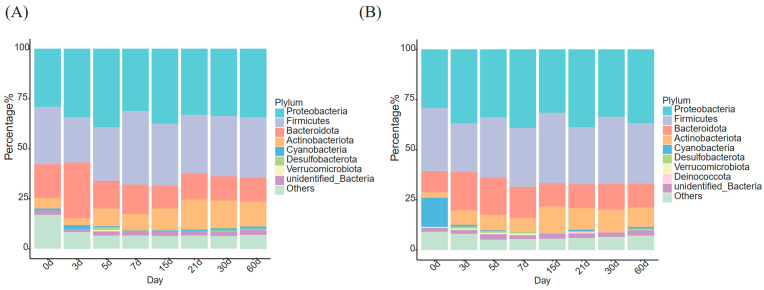
Dominant microbiota changes with DIM at the phylum level. (**A**) PC group; (**B**) MC group. PC: primiparous cows; MC: multiparous cows.

The phyla with a relative abundance greater than 0.5% were screened according to the time points for the PC group and MC group, and those whose relative abundance was less than 0.5% were classified as others (Figure 3). In both the PC and MC groups, Proteobacteria, Firmicutes, Bacteroidota, and Actinobacteriota were dominant phyla at each time point. Cyanobacteria was a dominant phylum at 7 time points (except 7 d) in the PC group, while it was a dominant phylum at 5 time points (except 7 d, 15 d, and 30 d) and showed greater abundance on 0 d in the MC group. Desulfobacterota was a dominant bacterium at 4 time points (3 d, 5 d, 30 d, and 60 d) in the PC group and 6 time points in the MC group (except 0 d and 30 d), accounting for a low but stable proportion (0.51–1.31%). From 0 d to 7 d, Verrucomicrobiota was a prevalent phylum in the MC group despite its different abundance order (0.51–0.74%). However, it was only dominant at 5 d in the PC group (0.60%). Deinococcuta was not a dominant bacterium in the PC group, but its relative abundance was greater than 0.5% at 21 d in the MC group (0.52%) (Table 2).

**Figure 4:**
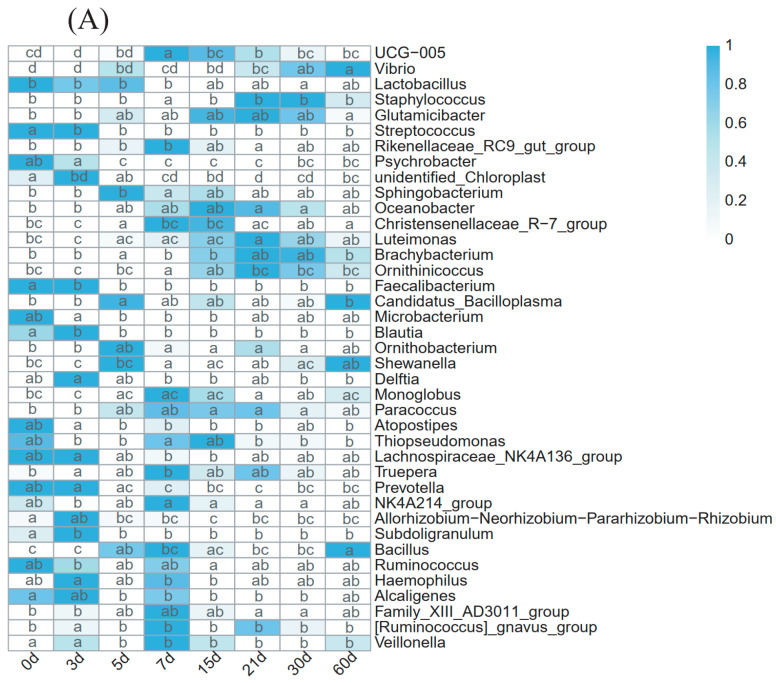

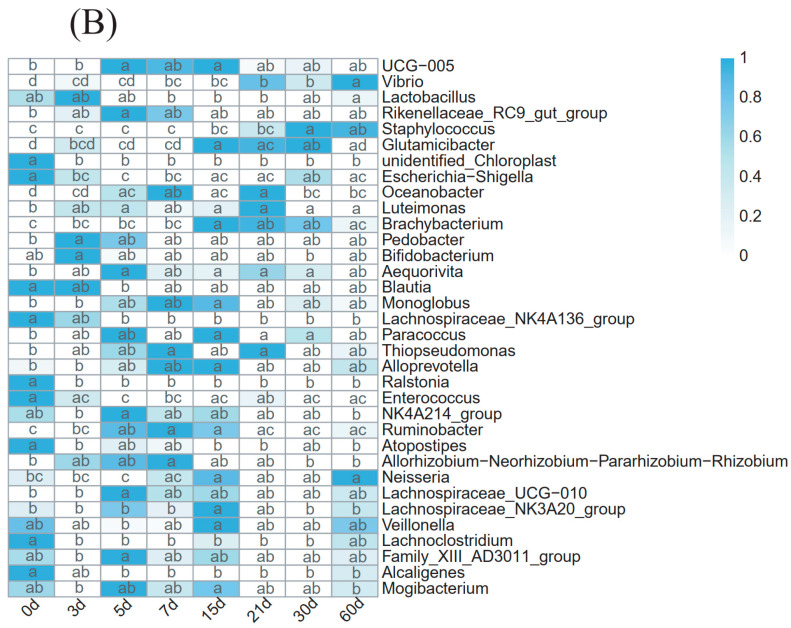
Shifts in the predominant genera (≥0.1% of the total sequences) in cow milk with DIM. (**A**) PC group; (**B**) MC group. Colors in boxes represent the abundances percentage of dominant genera. Different letters on each row denote significant differences among groups tested by the Tukey HSD test (*p* < 0.05). PC: primiparous cows; MC: multiparous cows.

At the genus level, a genus would be defined as the predominant bacterial taxa if its relative abundance was more than 0.1%. A genus was incorporated if its relative abundance was greater than 0.1% at all time points in the PC or MC group (Figure S3). In total, 65 taxa in the PC group and 62 taxa in the MC group were screened out in the milk microbial communities, and these predominant bacterial taxa accounted for over 60% (60.47–71.38%) of the total sequences, except for those of the MC group on 0 d (58.53%) and 7 d (58.85%). In order to analyze the variations in dominant genera at different time points, the Tukey HSD test was conducted for all genera at each time point. Significant differences (*p* < 0.05) in genus relative abundance at different time points were screened out and displayed in a heatmap (Figure 4). The PC and MC groups shared 56 predominant taxa, 35 of which shifted significantly with DIM in the PC group and 30 in the MC group, while the abundance of the remaining taxa was stable over time (Tables S2 and S3).

In total, 19 genera showed similar regulation in the two groups. High proportions of *Atopostipes*, *Blautia*, *Lactobacillus*, *Alcaligenes*, *Lachnospiraceae_NK4A136_group*, and *unidentified_Chloroplast* were found in the first 7 days. In contrast, *Luteimonas*, *Oceanobacter*, *Paracoccus*, *UCG-005*, *Thiopseudomonas*, *Monoglobus*, *Veillonella*, *Family_XIII_AD3011_group*, *NK4A214_group*, and *Rikenellaceae_RC9_gut_group* had high abundance in the medium term. Furthermore, the proportion of *Glutamicibacter* increased until 15 d and decreased slightly from then until 60 d. The relative abundance of *Vibrio* significantly increased from 30 d to 60 d, and the relative abundance of *Staphylococcus* significantly increased from 15 d in the PC group and 30 d in the MC group. In addition, the proportions of 15 genera (*Acinetobacter*, *Alistipes*, *Bacteroides*, *Parabacteroides*, etc.) remained stable in both groups. In the PC group, the proportions of 9 genera (*Aequorivita*, *Bifidobacterium*, *Enterococcus*, etc.) remained stable, while they fluctuated in the MC group, and the proportions of 11 genera (*Bacillus*, *Faecalibacterium*, *Microbacterium*, *Prevotella*, etc.) remained stable in the MC group, whereas they fluctuated in the PC group.

### 3.4. A Random Forest Model Correlating Milk Bacterial Taxonomic Biomarkers with DIM in Primiparous and Multiparous Cows

**Figure 5:**
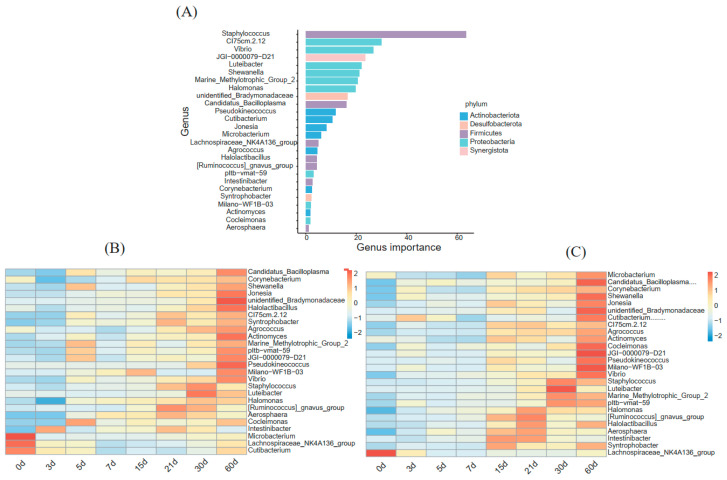
Biomarker genera in cow milk with DIM. (**A**) The list of the top 26 bacterial taxa at the genus level in cow milk in order of time-discriminatory importance. Relative abundance of the top 26 biomarker genera in the PC group (**B**) and the MC group (**C**). PC: primiparous cows; MC: multiparous cows.

To minimize the influence of parity, the relative abundance of milk genera was regressed using the Random Forest machine learning algorithm, thus establishing a model to correlate milk microbiota with DIM. This model explained 71.02% of the milk microbiota variance related to DIM. In order to evaluate the importance of bacterial genera as biomarker taxa with DIM, 10-fold cross-validation was performed with five repeats. The minimum cross-validation error was obtained when using 26 important genera (Figure 5A). Therefore, these 26 genera were defined as biomarker taxa in the model. The list of the top 26 bacterial taxa at the genus level in relation to DIM is shown in Figure 5B,C. Most biomarkers, such as *Cocleimonas*, *Pseudokineococcus*, *Aerosphaera*, etc. don’t showed high relative abundance until 15 d; more than half of them reached the highest abundance at 60 d.

### 3.5. Analysis of the Predominant Genera in Primiparous and Multiparous Cows

**Figure 6:**
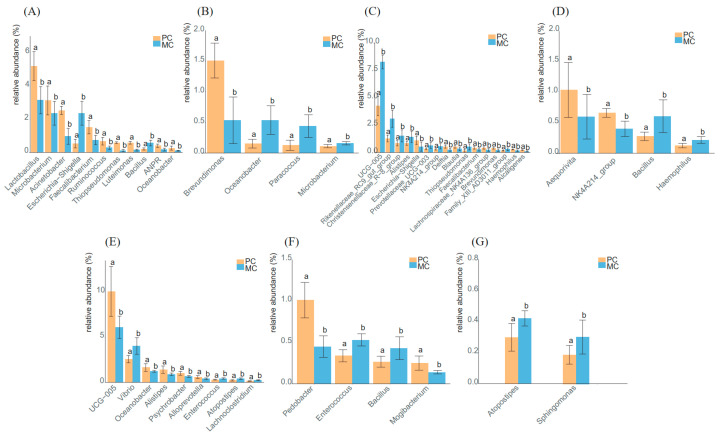
Dominant genera with significant differences in abundance in the two groups at each time point. (**A**–**G**): 0 d, 3 d, 5 d, 7 d, 15 d, 21 d, and 30 d. Different letters denote significant differences among groups tested by Wilcoxon test. Significance level: *p* < 0.05, test method: Wilcoxon test. PC: primiparous cows; MC: multiparous cows.

A Wilcoxon test was performed at each time point to identify the dominant genera in the two groups. The dominant genera with significant differences in abundance fluctuated from 0 d to 21 d (Figure 6A–F). A larger variety and higher abundance of genera were observed at 0 d (Figure 6A), 7 d (Figure 6C), and 15 d (Figure 6E), including *Lactobacillus*, *Microbacterium*, and *Acinetobacte* at 0 d, *UCG−005* at 7 d and 15 d, *Rikenellaceae_RC9_gut_group* at 7 d, and *Vibrio* at 15 d. These results revealed that the microbial structure in the colostrum and transitional milk was unstable, and significant differences were found between the primiparous and multiparous cow milk. From 21 d, the different genera between the two groups showed a low abundance, with a decrease in the number of different genera (Figure 6F). After 60 d, no difference in dominant genera was found, and the microbiota structure in the two groups tended to be similar (Figure 6G). These findings supported the hypothesis that the milk microflora of primiparous and multiparous cows gradually stabilized with DIM.

### 3.6. Co-Occurrence Networks of Characteristic Bacteria and the Components of Milk

**Figure 7:**
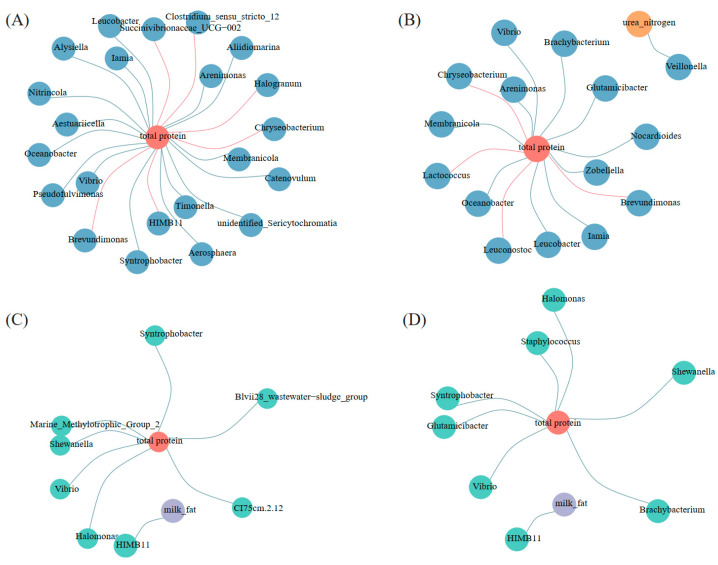
Co-occurrence correlation networks showed associations between genera in milk and milk components. (**A**) Co-occurrence correlation networks between specific genera in milk and milk components in the PC group; (**B**) Co-occurrence correlation networks between resident genus in milk and milk components in the PC group; (**C**) Co-occurrence correlation networks between specific genera in milk and milk components in the MC group; (**D**) Co-occurrence correlation networks between resident genus in milk and milk components in the MC group. Only strong (correlation coefficient *r* < −0.6 or *r* > 0.6) and significant (*p* < 0.05) correlations were chosen to be displayed in these networks. Red edge: positive correlation. Blue edge: negative correlation. PC: primiparous cows; MC: multiparous cows.

At each time point, a specific genus was defined by the following two conditions: (1) It only appeared in one group, or its relative abundance in one group was significantly different from that in the other group; (2) Its relative abundance was greater than 0.01%. A resident genus was characterized by a relative abundance of greater than 0.01% at each time point. A co-occurrence network was performed to explore the interactions between the milk genera and milk components (correlation coefficient *r* < −0.6 or *r* > 0.6, significant *p* < 0.05). To reduce the complexity of the networks, we only considered the relationship between the genera (the specific genera and the resident genera) in milk samples and the milk components (milk fat, total protein, and urea nitrogen) for network analysis (Figure 7). The concentration of milk components of cows at different time points was listed in Table 3.

In the PC group, total protein was significantly positively correlated (*r* > 0.6 and *p* < 0.05) with the relative abundances of six specific genera (*Succinivibrionaceae*, *Halogranum*, *Chryseobacterium*, *Brevundimonas*, etc.) (Figure 7A) and four resident genera (*Chryseobacterium*, *Lactococcus*, *Leuconostoc*, *Brevundimonas*, etc.) (Figure 7B), and was significantly negatively correlated (*r* < −0.6 and *p* < 0.05) with relative abundances of 16 specific genera (*Arenimonas*, *Aliidiomarina*, *Membranicola*, *Timonella*, etc.) (Figure 7A) and 10 resident genera (*Brachybacterium*, *Glutamicibacter*, *Nocardioides*, *Chryseobacterium*, etc.) (Figure 7B). In addition, urea nitrogen was significantly negatively correlated (*r* < −0.6 and *p* < 0.05) with the relative abundances of *Veillonella* (Figure 7B). In the MC group, total protein was significantly negatively correlated (*r* < −0.6 and *p* < 0.05) with the relative abundances of seven specific genera (*Syntrophobacter*, *Shewanella*, *Vibrio*, *Halomonas*, etc.) (Figure 7C) and seven resident genera (*Syntrophobacter*, *Glutamicibacter*, *Vibrio*, *Brachybacterium*, etc.) (Figure 7D). In addition, milk fat was significantly negatively correlated (*r* < −0.6 and *p* < 0.05) with the relative abundances of *HIMB11* (Figure 7C,D).

## 4. Discussion

This study used 16S rRNA high-throughput sequencing on the V4 region of bacterial communities in milk from primiparous and multiparous cows during different lactation stages. Great diversity has been reported in the intramammary bacterial community of humans, goats, and cows [15,24,25], which assisted the physiological development of the offspring [26]. The results of our study indicated that the lactation stage can influence the milk bacterial composition, and significant differences in the correlations between milk microorganisms and milk components were found between primiparous and multiparous cows.

The present study indicated that the alpha diversity at 0 d and 3 d were significantly different from those at other time points in the PC group, and remained relatively stable over time in the MC group. Meanwhile, the beta diversity showed an obvious accumulation of milk microbiota from 0 d to 3 d in the MC group. These findings may be attributed to the higher fluctuations in nutrient proportion in transitional and mature milk compared with colostrum, which was more obvious in the MC group.

In this study, Proteobacteria, Firmicutes, Bacteroidetes, and Actinobacteria were the most abundant phyla in the milk microbial community of dairy cows, regardless of parity and DIM, which was quite similar to the distribution pattern reviewed by Pang et al. [27], Rodrigues et al. [28], Derakhshani et al. [29], and Kable et al. [30]. Furthermore, Maity et al. also reported that the four phyla mentioned above were the main constituents of dairy cows’ milk microbiota, regardless of the mammary gland status [31]. Here, we observed an interesting phenomenon in both groups: although the abundance of these four phyla fluctuated within the sampling period, the total proportion remained stable at all timepoints except for 0 d (PC: 88.3~90.9%, MC: 87.5~91.3%). This indicated that the four phyla competed with each other for ecological niches. Proteobacteria were rich in microbiota diversity, and included a variety of non-pathogenic bacteria, although many Gram-negative environmental mastitis pathogens were also included [24]. Firmicutes and Bacteroides play an essential role in the health of ruminants. Members of Firmicutes produce butyrate and can degrade fiber and starch, which was connected to the intestinal health of the host [32]. Some members of Firmicutes, such as *Lactobacillus* and *Lactococcus*, were part of the normal milk microbiota. They can regulate the balance of intestinal flora and enhance the host’s immunity, and are considered beneficial probiotics [1]. In contrast, according to Oikonomu et al. [8] and Rodrigues et al. [29], some Gram-positive Firmicutes are contagious mastitis pathogens. *Bifidobacterium* is an important probiotic of Actinobacteria and is part of the physiological flora in human and animal intestines. However, additional studies are needed to better investigate the low abundance of Actinobacteria in the microbial community of milk.

Of the ten most average abundant genera, nine genera were found in both groups, including *Pseudomonas*, *UCG-005*, *Acinetobacter*, *Vibrio*, *Lactobacillus*, *Bacteroides*, *Serratia*, *Staphylococcus*, and *Glutamicibacter*. Similar to the results of this study, *Pseudomonas*, *Acinetobacter*, *Streptococcus*, *Bacteroidetes*, and *Lactobacillus* were predominant genera in cow milk [33,34,35,36]. Our results were slightly different from the above results, since the main pollution source of milk was the teat surface, and the factors affecting the microbial community on it were related to the dairy farm environment [37,38]. Despite strict disinfection during the sampling process, contamination of the teat skin was still possible, which could have affected the milk microbiota structure in this paper. However, the existence of strictly anaerobic bacteria, such as *Bacteroides*, *Lactobacillus*, and *Prevotella*, showed that our research was likely accurate, and not a result of contamination.

During the transition from colostrum to mature milk, *Acinetobacter*, *Bacteroidetes*, *Pseudomonas*, and *Serratia* showed relatively stable abundance in the two groups. *Acinetobacter* was frequently detected in raw milk and was considered a member of the core milk microbiota [27,30]. The role of *Acinetobacter* in milk was unclear, but *Acinetobacter* strains isolated from raw milk were associated with antibiotic resistance [39] and might help maintain a healthy status in cows [27]. *Bacteroides* are typically gut-associated microbes [40,41], and have been reported multiple times in cow milk microbiota [10,40,41,42]. Mtshali et al. (2022) suggested that its high abundance in milk samples might enhance calves’ immunity [41]. The reason for this might be that the nutritional components in transition and mature milk are relatively stable.

In addition to *Bacteroides*, *Alistipes*, *Parabacteroides*, *Prevotellaceae_UCG-003*, *Romboutsia*, and *Turicibacter* are also typically gut-associated microbes [41,43,44], and no significant difference in abundance was observed between each time point in the two groups. *UCG-005*, *Christensenellaceae_R-7_group*, *Family_XIII_AD3011_group*, *Monoglobus*, *NK4A214_group*, and *Rikenellaceae_RC9_gut_group* were also typically gut-associated microbes, and their abundance increased slightly from 5 d to 15 d. Recently, many scholars proposed the enteromammary pathway theory, stating that some microbes could move from the gut to the mammary gland via lymphatic and peripheral blood circulation [1,45,46]. Studies on mice showed that during pregnancy and lactation, the noticeable rise in intestinal epithelial permeability enhanced the possibility of intestinal bacterial translocation [47]. Similar bacterial translocation was also observed in cows. Luo et al. reported that the changes in hormone levels in cows during late pregnancy and lactation increased the permeability of the intestinal mucosal barrier, allowing bacteria to penetrate through the intestine. The bacteria were then taken up by lymphocytes and spread to the udder [48]. Similar studies also showed that the number of intestinal microorganisms transferred to other sites through dendritic cells during pregnancy or lactation may increase due to changes in the secretion of related hormones and the maternal physiological status [49]. Dairy cows undergo tremendous metabolic changes in the early lactation from 5 d to 15 d postpartum [50], which might explain the fluctuations in abundance of the above typically gut-associated microbes in milk. In addition, pathogenic bacteria could also reach the udder through the enteromammary pathway [51]. *Vibrio* is an enteric pathogenic bacterium [52]. Its increasing abundance with lactation time in the two groups might also be due to bacterial translocation.

Furthermore, a higher abundance of *Lactobacilli* was found during the first 5 days in the PC group and the first 3 days in the MC group (Figure 4). Its abundance in the PC group was significantly higher than that in the MC group at 0 d (Figure 6A). *Lactobacillus* is one of the most common lactic acid bacteria in milk [41]. Some *Lactobacillus* spp. can inhibit major mastitis pathogens such as *Escherichia* and *Serratia* [8]. *Lactobacillus* can also produce lactate and participate in lactate metabolism, establishing an effective trophic chain and avoiding lactic acid accumulation [53,54]. Similar to *Lactobacillus*, some genera showed higher abundance in the colostrum stage in the two groups, such as *Alcaligenes*, *Streptococcus*, *Veillonella*, *Blautia*, etc. In addition to nutritional composition changes over the transition period, hormonal and metabolic changes are also observed in cows [11,55], which may contribute to the differences in genera in colostrum. The alpha diversity also validated that the differences in milk diversity between the first three days and that of other time points were more obvious in the PC group (Figure 1).

Interestingly, a potential relationship between *Lactobacillus* and *Glutamicibacter* has recently been reported [56,57]. *Glutamicibacter* belongs to the Micrococcaceae family [56], which can produce short-chain fatty acids and may play a beneficial role in protecting the mucosal barrier and stimulating the host immune response [57]. Research on microorganisms interacting with the intestinal immune system found that Micrococcaceae and *Lactobacillus* were the most abundant families in meconium [58], while other studies demonstrated that the microbiota composition of meconium resembles that of colostrum [59,60]. Additionally, in the two groups, *Lactobacillus* don’t show higher relative abundance from 0 d to 3 d, and decreased with lactation time until 5 d. This was different from the significant increase in the proportions of *Glutamicibacter* from 15 d to 30 d. Thus, additional investigations were required to clarify the roles of these two genera in milk.

*Staphylococcus* was not only the dominant genera in the two groups, but also the DIM biomarker with the highest degree of interpretation analysis by the random forest algorithm. *Staphylococcus*, especially *S. aureus* and coagulase-negative *Staphylococcus*, was among the most prevalent genera in studies on both human and cow milk microbiota [1,8,29,35,61,62,63,64]. *S. aureus* is traditionally considered a major mastitis-related pathogen [16,65]. Nevertheless, some non-aureus species, such as *S. chromogens*, *S. simulans*, *S. xylosus*, etc., are often detected in bovine milk and are related to the prevention of infection of mastitis pathogens in milk by producing bacteriocin [66]. Moreover, as facultative anaerobes, *Staphylococcus* can participate in lactate metabolism, colonize the gastrointestinal tract (GIT), and contribute to the colonization of strict anaerobes by consuming O_2_ [67,68,69]. In addition, most of these biomarkers deduced by random forest algorithm began to accumulate since 15 d (Figure 5B,C), which also reflected the microbial composition of milk at the early stage of lactation to a certain extent. However, the potential relationship between the above biomarkers obtained in this study and breast health or disease requires further study.

Across the transition period, cows begin to lactate and go into a state of negative energy balance (NEBAL), which reaches a nadir during the first 2 weeks post-calving and will greatly affect animal health, milk yield, and subsequent fertility [13]. At present, other studies have reported that the microbial composition of the rumen, colostrum, and uterus was different between primiparous and multiparous cows in the transition period [10,13,14]. Nevertheless, there is currently a lack of research investigating the longitudinal changes in the milk microbiota of primiparous and multiparous cows. Our results demonstrated no significant differences in bacterial diversity between primiparous cows and multiparous cows. However, in the two groups of milk samples, there were more genera with differences in abundance at 0 d, 5 d, and 15 d, which were also higher than that of genera with differences at other time points. Notably, most of the different genera between the two groups, such as *Microbacterium*, *Faecalibacterium*, *UCG−005*, *Rikenellaceae_RC9_gut_group*, etc., were also typically gut-associated microbes. One hypothesis is the “enteromammary pathway theory”, in addition to the changes experienced by primiparous cows after first calving. These changes also confirmed that the differences between the two groups gradually decreased over time, until 60 d after delivery, at which point the microorganisms of the two groups tended to assimilate.

Microbial networks are a powerful and popular tool for investigating microbial communities. Figure 7 displays more genera associated with total protein in the primary group, including both specific genera and resident genera. Only the primary group contained genera that were positively correlated with total protein. Networks may be indicative of ecological characteristics [70], and the difference in the correlation between genera and total protein in the two groups may be related to the greater physiological fluctuation of the primiparous cows after calving. The potential relationship between genera and lactoprotein can be further analyzed in future research.

Overall, milk microbiota is a complex microbial community with abundant diversity and multifaceted biological effects [1]. Many strictly anaerobic bacteria, such as *Bacteroides*, *Lactobacillus*, and *Prevotella* were detected in our study and suggested that the microbiota in milk is not only related to the external environment. The high abundance of typically gut-associated microbes in the two groups confirmed that a part of the milk flora originated from the gastrointestinal tract, and this portion of gut-related bacteria would enter the breast, promoted by hormonal changes during and after pregnancy [11,51]. In the two groups, both the fluctuations in lactation time and the differences in the abundance of some typically gut-associated microbes showed that the physiological changes of dairy cows affect the diversity of milk microbial communities.

## 5. Conclusions

In total, 96 samples of primiparous and multiparous cows’ milk were collected from the day of parturition to 60 days postpartum, and 16S rRNA gene sequencing of the V4 region was performed to systematically characterize the milk microbiome by utilizing high-throughput sequencing. Proteobacteria, Firmicutes, Bacteroidota, and Actinobacteriota were the dominant phyla in the two groups at every different time point. Additionally, the abundance of some dominant genera during different lactation stages fluctuated with DIM; a greater difference in milk microbiota was found between primiparous and multiparous cows during early lactation (0 d–15 d). The difference gradually decreased from 21 d, until it disappeared at 60 d. The specific microorganisms selected in this study that were related to milk proteins can provide targeted bacterial information for improving the total protein content in milk, but further verification will be needed in combination with in vitro culturomics and model animals in the future.

## Figures and Tables

**Table 1:** Ingredients and chemical composition of dietary treatments of the experimental dairy cows.

Items	Value
Ingredient	%
Corn silage	39.52
Ground corn	21.83
Alfalfa hay	7.45
Cottonseed	3.81
DDGS (distiller dried grains with solubles)	3.41
Brewers grain	10.77
Soybean meal	5.34
Cottonseed protein	1.37
Soybean hulls	2.98
Cottonseed meal	0.85
Premix^2^	1.17
Limestone	0.7
Choline	0.05
Soda	0.75
Total	100
Nutrient levels	
Dry Matter in Dairy Ration (DM, %)	57.47
NEL3, MJ/kg	6.72
Crude Protein (CP, %)	14.80
Crude Fat (EE, %)	3.77
Crude Fiber (CF, %)	15.27
Neutral Detergent Fiber (NDF, %)	34.46
Acid detergent fiber (ADF, %)	21.34
Ash, %	4.85
Ca, %	0.52
P, %	0.37

**Table 2:** Average relative abundance percentage of dominant microbiota at each time point (phylum level).

Group	Phylum	0 d	3 d	5 d	7 d	15 d	21 d	30 d	60 d
PC	Proteobacteria	28.93	34.32	39.16	31.14	37.41	33.09	33.65	34.30
	Firmicutes	28.88	22.55	26.82	36.86	30.95	29.27	30.23	30.30
	Actinobacteriota	5.30	3.58	8.88	7.62	11.06	15.19	13.73	12.59
	Bacteroidota	16.79	27.86	13.83	14.61	11.45	12.91	12.10	11.85
	unidentified_Bacteria	2.18	1.50	2.19	2.58	2.08	2.22	2.20	2.50
	Cyanobacteria	0.97	1.86	0.64	0.44	0.52	0.77	1.19	0.87
	Desulfobacterota	0.34	0.22	1.31	0.65	0.48	0.45	0.50	0.66
	Verrucomicrobiota	0.39	0.34	0.60	0.49	0.48	0.35	0.36	0.34
MC	Proteobacteria	29.19	36.87	33.71	39.13	31.56	38.79	33.58	36.71
	Firmicutes	31.60	24.08	30.11	29.44	35.30	28.45	33.57	30.31
	Bacteroidota	10.42	19.26	18.61	15.40	11.45	11.99	12.67	11.78
	Actinobacteriota	2.75	7.30	7.72	6.95	13.03	10.65	11.46	9.79
	unidentified_Bacteria	1.90	1.99	2.60	1.92	2.49	2.24	2.18	2.50
	Cyanobacteria	14.47	0.82	0.51	0.44	0.31	0.76	0.46	0.87
	Desulfobacterota	0.49	0.96	0.74	0.90	0.51	0.57	0.47	0.72
	Verrucomicrobiota	0.51	0.64	0.69	0.74	0.29	0.32	0.30	0.34
	Deinococcota	0.07	0.24	0.31	0.29	0.31	0.52	0.26	0.28

PC: primiparous cows; MC: multiparous cows.

**Table 3:** The concentration of milk components of cows at different time points.

Group	Milk Component	0 d	3 d	5 d	7 d	15 d	21 d	30 d	60 d
PC	Milk Fat (%)	4.41 ± 0.72	2.38 ± 1.21	5.11 ± 0.861	5.19 ± 0.79	4.45 ± 0.83	4.58 ± 0.52	4.06 ± 1.15	3.72 ± 0.09
	Total Protein (%)	14.59 ± 1.14	6.29 ± 2.54	3.40 ± 0.26	3.27 ± 0.17	3.29 ± 0.22	3.03 ± 0.16	3.04 ± 0.28	3.05 ± 0.25
	Urea Nitrogen (mg/dL)	16.98 ± 3.12	13.95 ± 3.75	12.20 ± 3.35	12.63 ± 1.97	11.18 ± 3.22	11.16 ± 2.58	11.93 ± 2.82	12.17 ± 1.28
MC	Milk Fat (%)	4.16 ± 0.49	3.24 ± 1.52	5.39 ± 0.63	5.27 ± 0.39	4.82 ± 0.36	4.97 ± 0.25	3.84 ± 0.07	3.99 ± 0.50
	Total Protein (%)	16.56 ± 1.62	4.29 ± 1.32	4.00 ± 0.42	3.73 ± 0.18	3.14 ± 0.17	3.14 ± 0.19	3.10 ± 0.11	3.14 ± 0.28
	Urea Nitrogen (mg/dl)	15.11 ± 4.11	13.13 ± 2.22	12.36 ± 2.49	12.31 ± 2.99	12.29 ± 2.66	12.71 ± 2.89	13.07 ± 2.36	14.20 ± 2.82

PC: primiparous cows; MC: multiparous cows.

## Data Availability

Data will be made available upon request to the corresponding author.

## Supplementary Materials

The following supporting information can be downloaded at: https://www.mdpi.com/article/10.3390/ani13121923/s1, Figure S1: Shannon diversity sparse curves; Figure S2: The differences in microbiota structure of milk samples with DIM; Figure S3: Dominant microbiota changes with DIM at the genus level. Table S1: Sample information, microbial diversity, and sequence abundance at the genus level; Table S2: Comparison of the predominant genera (relative abundance ≥ 0.1%) in milk samples in the PC group at each time point; Table S3: Comparison of the predominant genera (relative abundance ≥0.1%) in milk samples in the MC group at each time point.
