# Supplementary material for: Longitudinal Changes in Milk Microorganisms in the First Two Months of Lactation of Primiparous and Multiparous Cows
Source: Animals (Basel). 2023 Jun 8;13(12):1923. doi: 10.3390/ani13121923 (PMC10294890; doi:10.3390/ani13121923)
Supplement: Supplementary file 1 [file animals-13-01923-s001.zip › animals-2333653-supplementary.pdf]

animals

Article

Longitudinal Changes in Milk Microorganisms in the First Two Months of Lactation of Primiparous and Multiparous Cows

Huan Zhu [1,2,3], Renfang Miao [1,2], Xinxu Tao [1,2], Jianhao Wu [4], Licheng Liu [5], Jiachen Qu [1,2], Hongzhi Liu [1,2], Yanting Sun [6], Lingyan Li [1,2] and Yongli Qu [1,2,*]

[1] Heilongjiang Key Laboratory of Efficient Utilization of Feed Resources and Nutrition Manipulation in Cold Region, College of Animal Science and Veterinary Medicine, Heilongjiang Bayi Agricultural University, No. 5 Xinyang Road, Daqing 163319, China; zhuhuan1982@sina.com (H.Z.); M_Leslie@163.com (R.M.); txx09090416@163.com (X.T.); qjc990313@163.com (J.Q.); hongzhiliu2023@163.com (H.L.); llytiger@163.com (L.L.)
[2] Key Laboratory of Low-Carbon Green Agriculture in Northeastern China, Ministry of Agriculture and Rural Affairs P. R. China, Heilongjiang Bayi Agricultural University, No. 5 Xinyang Road, Daqing 163319, China
[3] College of Science, Heilongjiang Bayi Agricultural University, No. 5 Xinyang Road, Daqing 163319, China
[4] Bright Farming Co., Ltd., No. 1518, West Jiangchang Road, Shanghai 200436, China; wujianhao@brightdairy.com
[5] Institute of Animal Husbandry and Veterinary Medicine, Heilongjiang Academy of Agricultural Reclamation, No. 101 Xiangfu Road, Herbin 150038, China; lichengliu2023@163.com
[6] School of Civil Engineering, Xi'an University of Architecture & Technology, No. 99 Yanta Road, Xi'an 710064, China; sunyanting98@163.com
* Correspondence: ylqu007@126.com; Tel.: +86-138-3696-1030

(A)

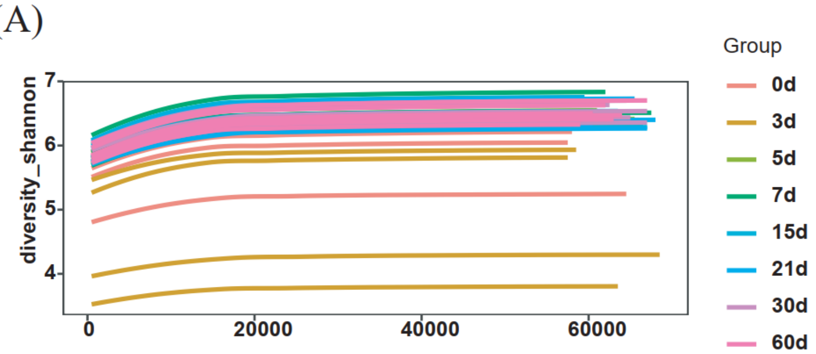

(B)

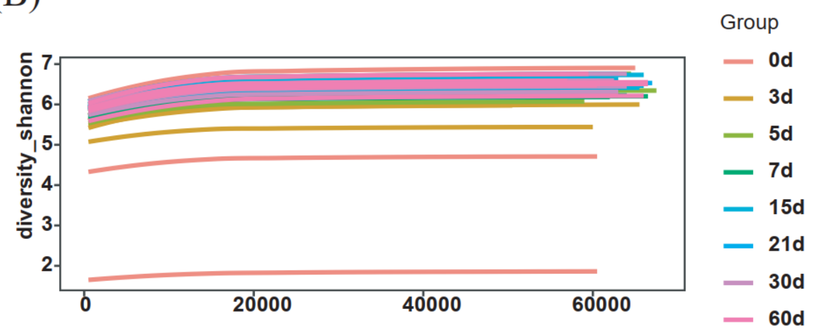

Figure S1. Shannon diversity sparse curves. (A) PC group, (B) MC group. PC: primiparous cows; MC: multiparous cows.

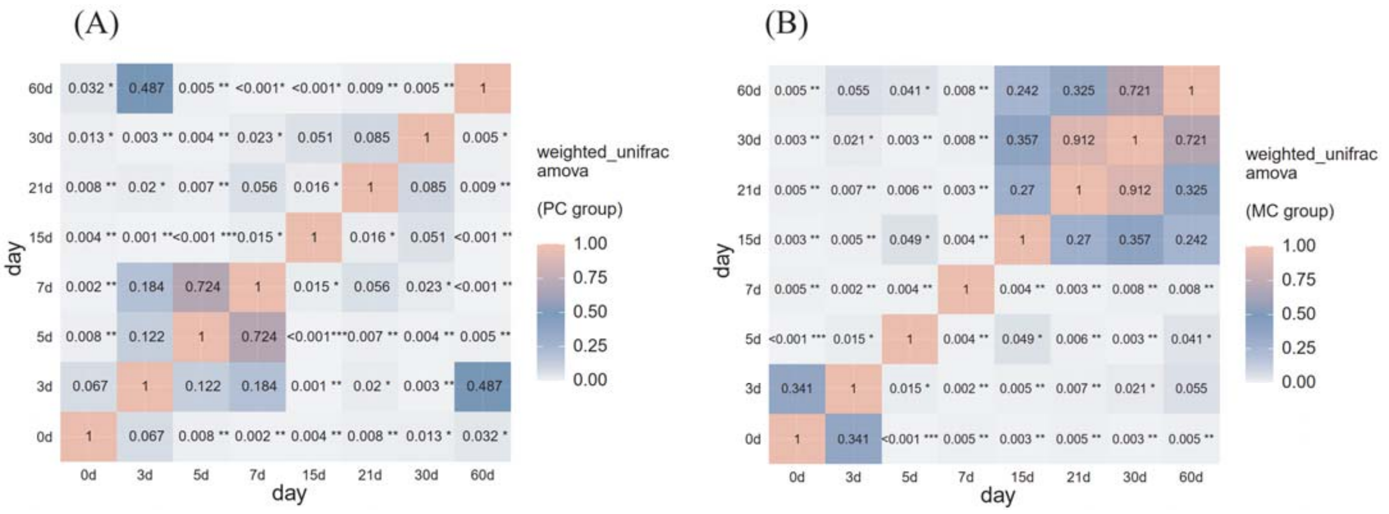

Figure S2. The differences in microbiota structure of milk samples with DIM. (A) PC group, (B) MC group. PC: primiparous cows; MC: multiparous cows.

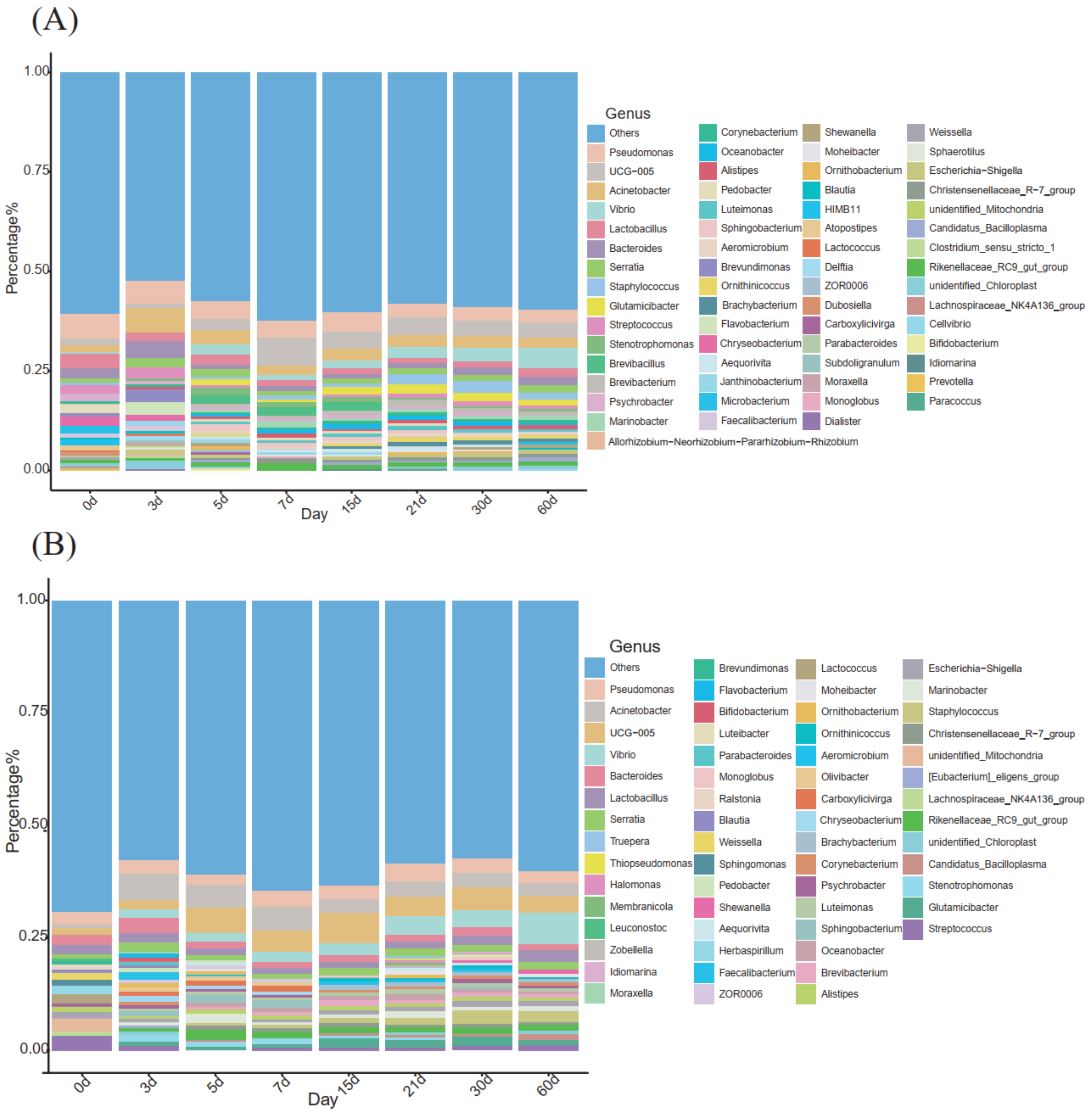

Figure S3. Dominant microbiota changes with DIM at the genus level. (A) PC group, (B) MC group. PC: primiparous cows; MC: multiparous cows.

Table S1. Sample information, microbial diversity, and sequence abundance at genus level.

Group	Day	Sample	Effective Tags	Number of Reads	Shannon	Chao1	Observed_species
	0d	PCRA1	59,598	92,346	8.705	2998.055	2717
	0d	PCRA2	61,093	98,013	8.946	3459.565	3104
	0d	PCRA4	67,951	109,063	9.218	3163.902	2963
	0d	PCRA6	65,925	107,490	7.551	2615.811	2411
	3d	PCRB1	69,699	113,625	6.19	2207.457	1994
	3d	PCRB2	64,486	104,875	5.478	1661.518	1489
	3d	PCRB4	60,909	100,422	8.546	2749.044	2453
	3d	PCRB5	60,046	101,963	8.367	3054.115	2729
	3d	PCRB6	67,113	101,201	9.2	2514.462	2391
	5d	PCRC1	61,834	103,155	9.354	3984.349	3448
	5d	PCRC2	66,707	108,181	9.226	4030.693	3566
	5d	PCRC3	60,989	100,892	9.105	4083.787	3578
	5d	PCRC4	62,821	104,022	9.418	4418.669	3779
	5d	PCRC5	67,478	101,247	9.228	4421.911	3863
	5d	PCRC6	60,387	97,422	9.136	4215.735	3641
	7d	PCRD1	61,872	98,931	9.407	3576.059	3181
	7d	PCRD2	67,966	81,416	9.206	3687.048	3300
	7d	PCRD3	61,832	103,540	9.551	4062.33	3562
	7d	PCRD4	69,219	110,735	9.37	4186.346	3634
	7d	PCRD5	64,006	101,812	9.033	3663.343	3275
	7d	PCRD6	63,594	99,105	9.835	4203.342	3742
	15d	PCRE1	68,234	106,530	9.048	3668.313	3231
	15d	PCRE2	65,240	106,460	9.406	3957.017	3496
	15d	PCRE3	62,668	101,596	9.206	3914.091	3382
	15d	PCRE4	67,912	112,569	9.101	3776.621	3341
	15d	PCRE5	61,526	99,663	9.72	4153.081	3676
	15d	PCRE6	60,030	92,919	9.144	3870.035	3341
	21d	PCRF1	61,774	99,916	9.099	3678.864	3186
	21d	PCRF2	69,615	110,235	9.21	4180.679	3660
	21d	PCRF3	68,363	105,745	9.018	3727.805	3261
	21d	PCRF4	62,224	101,429	9.542	4139.637	3611
	21d	PCRF5	66,966	110,240	9.23	3986.732	3520
	21d	PCRF6	67,311	113,108	9.681	4302.145	3766
	30d	PCRG1	68,929	106,077	9.146	4154.44	3653
	30d	PCRG2	63,390	99,561	9.196	3865.345	3337
	30d	PCRG3	64,137	103,791	9.548	4240.413	3721
	30d	PCRG4	67,171	109,673	9.258	4159.343	3649
	30d	PCRG5	66,645	107,210	9.639	4405.288	3853
	30d	PCRG6	68,987	115,187	9.406	4157	3662
	60d	PCRH1	60,307	95,727	9.102	3784.641	3263
	60d	PCRH2	64,110	103,444	9.536	4321.176	3782
	60d	PCRH3	67,243	112,296	9.307	4511.898	3887
	60d	PCRH4	69,535	110,458	9.64	4695.289	4097
	60d	PCRH5	65,660	108,731	9.217	4359.732	3718
PC	60d	PCRH6	62,426	100,221	9.344	4339.608	3793
	0d	MCRA1	61,884	97,016	6.782	2363.049	2157
	0d	MCRA2	69,312	111,178	9.937	5101.228	4613
MC	0d	MCRA3	64,347	103,019	9.5	4602.687	4006
	0d	MCRA4	60,861	101,865	9.292	4692.852	4085
	0d	MCRA5	61,202	94,264	9.136	4327.083	4042
	0d	MCRA6	51,724	84,039	9.088	4192.651	4091

3d	MCRB1	53,800	92,688	8.604	2918.155	2694
3d	MCRB2	61,396	100,263	7.84	2172.125	2024
3d	MCRB3	66,869	103,193	8.626	3840.136	3314
3d	MCRB4	65,573	105,586	8.929	4143.879	3631
3d	MCRB5	62,214	100,371	8.944	3966.519	3443
3d	MCRB6	64,282	104,237	9.541	4335.238	3831
5d	MCRC1	66,680	105,158	9.723	4596.987	4031
5d	MCRC2	60,064	101,916	8.74	3280.858	2900
5d	MCRC3	68,850	100,666	9.132	4117.735	3616
5d	MCRC4	60,435	84,337	9.189	3559.286	3141
5d	MCRC5	62,340	104,857	9.338	3625.08	3188
5d	MCRC6	62,218	101,416	9.281	3800.172	3357
7d	MCRD1	64,649	101,979	8.898	2932.051	2558
7d	MCRD2	65,880	102,556	8.959	4043.041	3512
7d	MCRD3	67,799	106,304	8.926	3574.754	3162
7d	MCRD4	65,409	104,732	9.377	3672.866	3319
7d	MCRD5	62,443	100,587	9.094	3813.168	3331
7d	MCRD6	63,017	98,851	9.298	3687.24	3251
15d	MCRE1	64,562	109,317	9.539	4099.228	3567
15d	MCRE2	66,639	105,938	9.342	3867.182	3415
15d	MCRE3	61,569	102,513	9.542	4004.944	3550
15d	MCRE4	67,525	110,401	9.303	3921.355	3470
15d	MCRE5	67,053	110,601	9.23	3969.047	3476
15d	MCRE6	67,566	111,006	9.684	4368.639	3863
21d	MCRF1	65,398	109,245	9.371	3925.805	3461
21d	MCRF2	61,369	101,915	9.498	4121.28	3621
21d	MCRF3	63,899	99,556	9.265	3920.41	3399
21d	MCRF4	68,637	108,717	9.399	3975.911	3530
21d	MCRF5	64,852	108,093	9.715	4314.662	3829
21d	MCRF6	61,154	102,723	9.409	3910.996	3445
30d	MCRG1	64,541	106,849	9.486	3963.426	3578
30d	MCRG2	61,626	99,742	9.337	4026.086	3544
30d	MCRG3	65,787	110,339	9.276	3948.435	3493
30d	MCRG4	65,682	109,470	9.729	4393.666	3916
30d	MCRG5	62,499	103,945	9.356	3843.497	3444
30d	MCRG6	64,222	99,689	9.082	3693.299	3312
60d	MCRH1	62,239	102,288	9.319	4302.146	3756
60d	MCRH2	68,282	111,498	9.363	4429.816	3860
60d	MCRH3	68,457	107,728	8.935	4333.779	3819
60d	MCRH4	61,564	97,447	9.721	4736.136	4070
60d	MCRH5	68,648	110,556	9.421	4530.696	3909
60d	MCRH6	60,030	97,167	9.468	4123.962	3635

PC: primiparous cows; MC: multiparous cows; Effective Tags: the final Tags sequence for subsequent analysis after filtering chimera; Number of reads: PE reads of original offline

Table S2. Comparison of the predominant genera (relative abundance ≥0.1%) in milk samples in the PC group at each time point. Significance level: $P < 0.05$, test method: Tukey HSD test.

Genus	0d	3d	5d	7d	15d	21d	30d	60d
UCG-005	0.0230cd	0.0139d	0.0425bd	0.1033a	0.0610bc	0.0648b	0.0575bc	0.0551bc
Vibrio	0.0073d	0.0047d	0.0338bd	0.0208cd	0.0257bd	0.0407bc	0.0494ab	0.0713a
Lactobacillus	0.0519b	0.0319b	0.0378b	0.0199b	0.0241ab	0.0167ab	0.0211a	0.0332ab
Staphylococcus	0.0095b	0.0060b	0.0122b	0.0131a	0.0146b	0.0339b	0.0411b	0.0262b
Glutamicibacter	0.0013b	0.0022b	0.0186ab	0.0111ab	0.0261ab	0.0343ab	0.0297ab	0.0215a
Streptococcus	0.0333a	0.0407b	0.0094b	0.0064b	0.0091b	0.0129b	0.0161b	0.0134b
Rikenellaceae_RC9_gut_group	0.0105b	0.0067b	0.0127b	0.0269b	0.0133ab	0.0131a	0.0139ab	0.0152ab
Psychrobacter	0.0273ab	0.0118a	0.0095c	0.0083c	0.0101c	0.0109c	0.0093bc	0.0065bc
unidentified_Chloroplast	0.0135a	0.0268bd	0.0076ab	0.0037cd	0.0053bd	0.0089d	0.0141cd	0.0107bc
Sphingobacterium	0.0033b	0.0047b	0.0238b	0.0156a	0.0143ab	0.0116ab	0.0070ab	0.0059ab
Oceanobacter	0.0026b	0.0015b	0.0089ab	0.0144ab	0.0164ab	0.0168a	0.0146a	0.0083ab
Christensenellaceae_R-7_group	0.0061bc	0.0048c	0.0084a	0.0173bc	0.0116bc	0.0108ac	0.0107ab	0.0104a
Luteimonas	0.0058bc	0.0036c	0.0091ac	0.0101ac	0.0107ac	0.0143a	0.0129ab	0.0116ab
Brachybacterium	0.0012b	0.0017b	0.0058a	0.0050b	0.0113b	0.0167ab	0.0152ab	0.0129b
Ornithinicoccus	0.0032bc	0.0016c	0.0074bc	0.0055a	0.0102ab	0.0156bc	0.0129bc	0.0112bc
Faecalibacterium	0.0152a	0.0183b	0.0042b	0.0031b	0.0033b	0.0048b	0.0064b	0.0070b
Candidatus_Bacilloplasma	0.0024b	0.0014b	0.0103a	0.0060ab	0.0088ab	0.0075ab	0.0092ab	0.0158b
Microbacterium	0.0313ab	0.0011a	0.0023b	0.0015b	0.0022b	0.0045ab	0.0063ab	0.0086ab
Blautia	0.0086a	0.0114b	0.0051b	0.0038b	0.0043b	0.0054b	0.0070b	0.0072b
Ornithobacterium	0.0042b	0.0032b	0.0125ab	0.0057a	0.0058a	0.0098a	0.0073a	0.0037ab
Shewanella	0.0020bc	0.0011c	0.0107bc	0.0031a	0.0032ac	0.0068ab	0.0082ac	0.0125ab
Delftia	0.0039ab	0.0146a	0.0053ab	0.0026b	0.0050b	0.0038ab	0.0041b	0.0044b
Monoglobus	0.0035bc	0.0021c	0.0041ac	0.0084ac	0.0061ac	0.0062a	0.0061ab	0.0065ac
Paracoccus	0.0012b	0.0013b	0.0055ab	0.0047ab	0.0059a	0.0072a	0.0062a	0.0054ab
Atopostipes	0.0158ab	0.0024a	0.0023b	0.0044b	0.0040b	0.0024b	0.0030ab	0.0027b
Thiopseudomonas	0.0058ab	0.0010b	0.0016b	0.0045a	0.0065ab	0.0055b	0.0058b	0.0049b

Lachnospiraceae_NK4A136_group	0.0081ab	0.0049a	0.0041ab	0.0026b	0.0028b	0.0031ab	0.0036ab	0.0050ab
Truepera	0.0040b	0.0012a	0.0039ab	0.0048b	0.0046ab	0.0063ab	0.0056ab	0.0037ab
Prevotella	0.0075ab	0.0058a	0.0035ac	0.0026c	0.0026bc	0.0028c	0.0030bc	0.0035bc
NK4A214_group	0.0040ab	0.0026b	0.0027ab	0.0066a	0.0041a	0.0037a	0.0032a	0.0031ab
Allorhizobium-Neorhizobium-Pararhizobium-Rhizobium	0.0040a	0.0091ab	0.0046bc	0.0019bc	0.0023c	0.0024bc	0.0021bc	0.0018bc
Subdoligranulum	0.0042a	0.0082b	0.0017b	0.0012b	0.0011b	0.0030b	0.0038b	0.0040b
Bacillus	0.0021c	0.0018c	0.0046ab	0.0027bc	0.0036ac	0.0027bc	0.0033bc	0.0055a
Ruminococcus	0.0067ab	0.0029b	0.0019ab	0.0022ab	0.0018a	0.0021ab	0.0021ab	0.0023ab
Haemophilus	0.0018ab	0.0038a	0.0027ab	0.0013b	0.0023b	0.0022ab	0.0022ab	0.0028ab
Alcaligenes	0.0037a	0.0041ab	0.0021b	0.0013b	0.0017b	0.0012b	0.0015b	0.0017ab
Family_XIII_AD3011_group	0.0013b	0.0015b	0.0016ab	0.0038ab	0.0025ab	0.0023a	0.0023a	0.0019ab
[Ruminococcus]_gnavus_group	0.0013b	0.0014a	0.0013b	0.0013b	0.0020b	0.0037b	0.0032b	0.0021b
Veillonella	0.0011a	0.0017a	0.0019b	0.0016b	0.0027b	0.0021b	0.0022b	0.0026b
Acinetobacter	0.0253a	0.0847a	0.0511a	0.0347a	0.0448a	0.0414a	0.0391a	0.0387a
Alistipes	0.0067a	0.0086a	0.0084a	0.0148a	0.0089a	0.0107a	0.0113a	0.0130a
Bacteroides	0.0373a	0.0568a	0.0166a	0.0215a	0.0132a	0.0217a	0.0268a	0.0299a
Brevundimonas	0.0139a	0.0461a	0.0038a	0.0021a	0.0029a	0.0020a	0.0020a	0.0028a
Clostridium_sensu_stricto_1	0.0032a	0.0089a	0.0070a	0.0057a	0.0060a	0.0050a	0.0047a	0.0049a
Corynebacterium	0.0112a	0.0073a	0.0080a	0.0092a	0.0118a	0.0113a	0.0112a	0.0123a
Flavobacterium	0.0063a	0.0457a	0.0074a	0.0062a	0.0038a	0.0047a	0.0035a	0.0026a
Parabacteroides	0.0089a	0.0066a	0.0020a	0.0018a	0.0017a	0.0019a	0.0025a	0.0028a
Prevotellaceae_UCG-003	0.0037a	0.0028a	0.0031a	0.0062a	0.0035a	0.0044a	0.0044a	0.0046a
Pseudomonas	0.0889a	0.0813a	0.0625a	0.0601a	0.0700a	0.0499a	0.0476a	0.0435a
Romboutsia	0.0041a	0.0042a	0.0056a	0.0067a	0.0065a	0.0051a	0.0047a	0.0050a
Serratia	0.0168a	0.0302a	0.0248a	0.0152a	0.0192a	0.0207a	0.0223a	0.0250a
Sphingomonas	0.0021a	0.0027a	0.0021a	0.0022a	0.0023a	0.0018a	0.0019a	0.0020a
Stenotrophomonas	0.0046a	0.0067a	0.0286a	0.0168a	0.0181a	0.0084a	0.0073a	0.0080a
Turicibacter	0.0013a	0.0021a	0.0021a	0.0022a	0.0023a	0.0020a	0.0016a	0.0016a

Aequorivita	0.0035a	0.0031a	0.0085a	0.0103a	0.0100a	0.0137a	0.0098a	0.0065a
Alloprevotella	0.0033a	0.0029a	0.0035a	0.0037a	0.0040a	0.0039a	0.0040a	0.0051a
Bifidobacterium	0.0066a	0.0063a	0.0076a	0.0056a	0.0052a	0.0062a	0.0062a	0.0064a
Enterococcus	0.0045a	0.0040a	0.0032a	0.0022a	0.0030a	0.0034a	0.0039a	0.0032a
Escherichia-Shigella	0.0053a	0.0132a	0.0111a	0.0066a	0.0098a	0.0087a	0.0125a	0.0144a
Lachnoclostridium	0.0024a	0.0026a	0.0017a	0.0013a	0.0014a	0.0017a	0.0019a	0.0021a
Lachnospiraceae_NK3A20_group	0.0018a	0.0055a	0.0024a	0.0035a	0.0037a	0.0041a	0.0035a	0.0030a
Mogibacterium	0.0014a	0.0028a	0.0019a	0.0029a	0.0034a	0.0025a	0.0022a	0.0021a
Pedobacter	0.0272a	0.0063a	0.0114a	0.0083a	0.0081a	0.0101a	0.0064a	0.0060a
UCG-002	0.0018a	0.0021a	0.0011a	0.0016a	0.0010a	0.0017a	0.0018a	0.0019a
Pseudoalteromonas	0.0043a	0.0010a	0.0016a	0.0010a	0.0030a	0.0042a	0.0038a	0.0019a

PC: primiparous cows.

Table S3. Comparison of the predominant genera (relative abundance ⩾0.1%) in milk samples in the MC group at each time point. Significance level: $P < 0.05$, test method: Tukey HSD test.

Genus	0d	3d	5d	7d	15d	21d	30d	60d
UCG-005	0.0245b	0.0286b	0.0826a	0.0681ab	0.1007a	0.0602ab	0.0722ab	0.0522ab
Vibrio	0.0025d	0.0247cd	0.0259cd	0.0321bc	0.0403bc	0.0571b	0.0547b	0.0994a
Lactobacillus	0.0314ab	0.0317ab	0.0214ab	0.0192b	0.0207b	0.0200b	0.0272ab	0.0381a
Rikenellaceae_RC9_gut_group	0.0084b	0.0124ab	0.0308a	0.0200ab	0.0189ab	0.0163ab	0.0196ab	0.0167ab
Staphylococcus	0.0066c	0.0061c	0.0082c	0.0091c	0.0161bc	0.0202bc	0.0406a	0.0329ab
Glutamicibacter	0.0012d	0.0120bcd	0.0105cd	0.0108cd	0.0293a	0.0236ac	0.0279ab	0.0153ad
unidentified_Chloroplast	0.0698a	0.0103b	0.0046b	0.0041b	0.0030b	0.0075b	0.0048b	0.0104b
Escherichia-Shigella	0.0236a	0.0108bc	0.0053c	0.0082bc	0.0128ac	0.0139ac	0.0199ab	0.0138ac
Oceanobacter	0.0010d	0.0054cd	0.0126ac	0.0155ab	0.0122ac	0.0183a	0.0103bc	0.0086bc
Luteimonas	0.0014b	0.0075ab	0.0099a	0.0083ab	0.0113a	0.0134a	0.0100a	0.0106a
Brachybacterium	0.0012c	0.0032bc	0.0045bc	0.0051bc	0.0152a	0.0114ab	0.0120ab	0.0087ac
Pedobacter	0.0014b	0.0115a	0.0105ab	0.0063ab	0.0073ab	0.0045ab	0.0060ab	0.0046ab
Bifidobacterium	0.0050ab	0.0121a	0.0070ab	0.0064ab	0.0055ab	0.0053ab	0.0044b	0.0057ab
Aequorivita	0.0011b	0.0051ab	0.0083a	0.0059ab	0.0074a	0.0082a	0.0081a	0.0054ab
Blautia	0.0104a	0.0085ab	0.0033b	0.0039ab	0.0057ab	0.0062ab	0.0060ab	0.0051ab
Monoglobus	0.0036b	0.0031b	0.0062ab	0.0074ab	0.0087a	0.0053ab	0.0076ab	0.0066ab
Lachnospiraceae_NK4A136_group	0.0163a	0.0069ab	0.0023b	0.0019b	0.0036b	0.0033b	0.0034b	0.0055b
Paracoccus	0.0023b	0.0044ab	0.0050ab	0.0040ab	0.0063a	0.0053a	0.0062a	0.0035ab
Thiopseudomonas	0.0011b	0.0031ab	0.0051ab	0.0067a	0.0046ab	0.0074a	0.0035ab	0.0050ab
Alloprevotella	0.0029b	0.0029b	0.0037ab	0.0049ab	0.0059a	0.0042ab	0.0045ab	0.0047ab
Ralstonia	0.0184a	0.0025b	0.0017b	0.0019b	0.0019b	0.0015b	0.0019b	0.0031b
Enterococcus	0.0065a	0.0045ac	0.0021c	0.0027bc	0.0043ac	0.0053ab	0.0035ac	0.0035ac
NK4A214_group	0.0033ab	0.0021b	0.0057a	0.0040ab	0.0049ab	0.0029ab	0.0038ab	0.0025b
Ruminobacter	0.0010c	0.0017bc	0.0044ab	0.0055a	0.0052a	0.0033ac	0.0036ac	0.0033ac
Atopostipes	0.0075a	0.0021b	0.0032ab	0.0034ab	0.0022b	0.0024b	0.0042ab	0.0022b
Allorhizobium-Neorhizobium-Pararhizobium-Rhizobium	0.0017b	0.0044ab	0.0043ab	0.0061a	0.0023ab	0.0022ab	0.0017b	0.0017b

Neisseria	0.0018bc	0.0020bc	0.0016c	0.0029ac	0.0038a	0.0035ab	0.0034ab	0.0039a
Lachnospiraceae_UCG-010	0.0011b	0.0013b	0.0039a	0.0030ab	0.0033ab	0.0023ab	0.0026ab	0.0024ab
Lachnospiraceae_NK3A20_group	0.0014b	0.0016b	0.0025b	0.0024b	0.0040a	0.0026ab	0.0025b	0.0022b
Veillonella	0.0021ab	0.0018ab	0.0015b	0.0022ab	0.0037a	0.0029ab	0.0021ab	0.0028ab
Lachnoclostridium	0.0042a	0.0019b	0.0013b	0.0017b	0.0024b	0.0023b	0.0020b	0.0025ab
Family_XIII_AD3011_group	0.0016ab	0.0012b	0.0029a	0.0022ab	0.0027ab	0.0023ab	0.0021ab	0.0016ab
Alcaligenes	0.0042a	0.0029ab	0.0010b	0.0013b	0.0012b	0.0016b	0.0013b	0.0018b
Mogibacterium	0.0014ab	0.0011b	0.0023ab	0.0021ab	0.0026a	0.0014ab	0.0016ab	0.0013b
Acinetobacter	0.0097a	0.0840a	0.0735a	0.0776a	0.0421a	0.0488a	0.0433a	0.0396a
Alistipes	0.0180a	0.0077a	0.0142a	0.0118a	0.0141a	0.0108a	0.0137a	0.0125a
Bacteroides	0.0370a	0.0187a	0.0240a	0.0208a	0.0214a	0.0234a	0.0267a	0.0190a
Brevundimonas	0.0107a	0.0053a	0.0020a	0.0022a	0.0024a	0.0023a	0.0025a	0.0029a
Clostridium_sensu_stricto_1	0.0067a	0.0044a	0.0049a	0.0046a	0.0070a	0.0049a	0.0045a	0.0053a
Corynebacterium	0.0059a	0.0094a	0.0067a	0.0064a	0.0101a	0.0107a	0.0109a	0.0120a
Flavobacterium	0.0048a	0.0119a	0.0036a	0.0038a	0.0024a	0.0031a	0.0056a	0.0031a
Parabacteroides	0.0074a	0.0135a	0.0021a	0.0018a	0.0020a	0.0020a	0.0022a	0.0027a
Prevotellaceae_UCG-003	0.0027a	0.0028a	0.0064a	0.0047a	0.0051a	0.0035a	0.0053a	0.0049a
Pseudomonas	0.0798a	0.0438a	0.0348a	0.0531a	0.0485a	0.0571a	0.0490a	0.0389a
Romboutsia	0.0055a	0.0036a	0.0054a	0.0048a	0.0057a	0.0045a	0.0045a	0.0044a
Serratia	0.0165a	0.0279a	0.0163a	0.0163a	0.0242a	0.0252a	0.0218a	0.0245a
Sphingomonas	0.0225a	0.0021a	0.0025a	0.0022a	0.0022a	0.0024a	0.0030a	0.0022a
Stenotrophomonas	0.0033a	0.0224a	0.0171a	0.0198a	0.0095a	0.0081a	0.0061a	0.0065a
Turicibacter	0.0043a	0.0014a	0.0020a	0.0018a	0.0024a	0.0015a	0.0015a	0.0020a
Bacillus	0.0056a	0.0049a	0.0052a	0.0060a	0.0032a	0.0043a	0.0045a	0.0062a
Christensenellaceae_R-7_group	0.0075a	0.0064a	0.0151a	0.0146a	0.0142a	0.0101a	0.0115a	0.0101a
Delftia	0.0040a	0.0052a	0.0024a	0.0043a	0.0048a	0.0043a	0.0040a	0.0042a
Faecalibacterium	0.0075a	0.0267a	0.0026a	0.0070a	0.0038a	0.0052a	0.0047a	0.0045a
Haemophilus	0.0017a	0.0069a	0.0015a	0.0022a	0.0027a	0.0027a	0.0025a	0.0037a

Microbacterium	0.0033a	0.0016a	0.0016a	0.0011a	0.0049a	0.0033a	0.0043a	0.0061a
Ornithobacterium	0.0013a	0.0140a	0.0097a	0.0047a	0.0051a	0.0093a	0.0070a	0.0058a
Prevotella	0.0075a	0.0065a	0.0031a	0.0029a	0.0030a	0.0023a	0.0030a	0.0047a
Psychrobacter	0.0101a	0.0108a	0.0079a	0.0089a	0.0066a	0.0068a	0.0131a	0.0076a
Ruminococcus	0.0028a	0.0019a	0.0023a	0.0025a	0.0024a	0.0019a	0.0021a	0.0024a
Streptococcus	0.0450a	0.0143a	0.0067a	0.0087a	0.0098a	0.0091a	0.0156a	0.0165a
UCG-009	0.0025a	0.0014a	0.0035a	0.0033a	0.0031a	0.0022a	0.0028a	0.0024a
Phascolarctobacterium	0.0022a	0.0039a	0.0023a	0.0015a	0.0011a	0.0010a	0.0011a	0.0011a

MC: multiparous cows.
